# The Anthelmintic Quassinoids Ailanthone and Bruceine a Induce Infertility in the Model Organism *Caenorhabditis elegans* by an Apoptosis-like Mechanism Induced in Gonadal and Spermathecal Tissues

**DOI:** 10.3390/molecules26237354

**Published:** 2021-12-03

**Authors:** Nicola Knetzger, Viktoria Bachtin, Susanne Lehmann, Andreas Hensel, Eva Liebau, Fabian Herrmann

**Affiliations:** 1 Institute for Pharmaceutical Biology and Phytochemistry (IPBP), University of Muenster, Pharma Campus, Corrensstrasse 48, D-48149 Muenster, Germany; nicola.knetzger@isc.fraunhofer.de (N.K.); v_bach02@uni-muenster.de (V.B.); s_lehm08@uni-muenster.de (S.L.); ahensel@uni-muenster.de (A.H.); 2 Department of Molecular Physiology, Institute of Animal Physiology, University of Muenster, Schlossplatz 8, D-48143 Muenster, Germany; liebaue@uni-muenster.de

**Keywords:** atomic force microscopy, ultramicrotomy, polyethylene glycol embedding, ultrastructural morphology, anthelmintic natural products, *Caenorhabditis elegans*, quassinoid, ailanthone, bruceine A

## Abstract

In continuation of the search for new anthelmintic natural products, the study at hand investigated the nematicidal effects of the two naturally occurring quassinoids ailanthone and bruceine A against the reproductive system of the model nematode *Caenorhabditis elegans* to pinpoint their anthelmintic mode of action by the application of various microscopic techniques. Differential Interference Contrast (DIC) and the epifluorescence microscopy experiments used in the presented study indicated the genotoxic effects of the tested quassinoids (c _ailanthone_ = 50 µM, c _bruceine A_ = 100 µM) against the nuclei of the investigated gonadal and spermathecal tissues, leaving other morphological key features such as enterocytes or body wall muscle cells unimpaired. In order to gain nanoscopic insight into the morphology of the gonads as well as the considerably smaller spermathecae of *C. elegans*, an innovative protocol of polyethylene glycol embedding, ultra-sectioning, acridine orange staining, tissue identification by epifluorescence, and subsequent AFM-based ultrastructural data acquisition was applied. This sequence allowed the facile and fast assessment of the impact of quassinoid treatment not only on the gonadal but also on the considerably smaller spermathecal tissues of *C. elegans*. These first-time ultrastructural investigations on *C. elegans* gonads and spermathecae by AFM led to the identification of specific quassinoid-induced alterations to the nuclei of the reproductive tissues (e.g., highly condensed chromatin, impaired nuclear membrane morphology, as well as altered nucleolus morphology), altogether implying an apoptosis-like effect of ailanthone and bruceine A on the reproductive tissues of *C. elegans*.

## 1. Introduction

**Figure 1:**
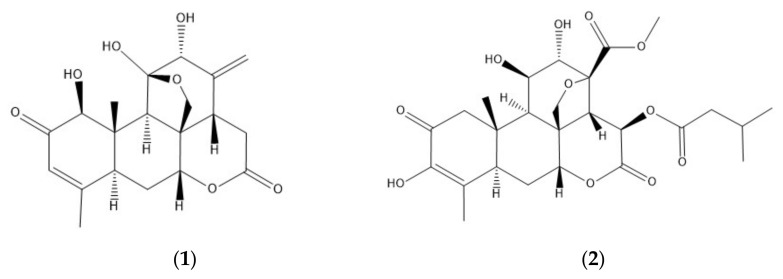
Structures of the tested quassinoids ailanthone (**1**) and bruceine A (**2**).

Infectious diseases caused by pathogenic helminths are responsible for a striking global health burden, especially in socioeconomically less developed countries all over the world [1,2]. In order to develop innovative therapeutic options against infectious helminths, ethnopharmacological approaches have proven to be of high relevance for the identification of new anthelmintic lead substances from natural origin [3,4,5]. In course of an ethnopharmacological study carried out recently, a quassinoid-rich hydroethanolic extract from *Ailanthus altissima* (Mill.) Swingle (Simaroubaceae) was identified to exert promising anthelmintic activity [6]. The family Simaroubaceae is altogether known for a variety of genera investigated for their medicinal properties (e.g., the genus *Eurycoma* for antimicrobial, anti-plasmodial or anti-fungal properties [7], the genus *Picrasma* for anti-inflammatory, anti-cancer or anti-viral effects [8] or the genus *Quassia* for anti-plasmodial effects [9]). In general, quassinoids are a class of terpenoid-like natural products and are commonly known for a diversity of promising biological activities [10,11,12,13]. These degraded triterpenes can be structurally diversified by having either a C-18, C-19, C-20, C-22, or a C-25 skeleton [14], and the majority of quassinoids exerting interesting bioactivities typically contains a δ-lactone and a methylenoxy bridge between C-8 and C-11 (e.g., ailanthone, Figure 1(**1**)) or C-8 and C-13 (e.g., bruceine A, Figure 1(**2**)) [15]. The variable substitution pattern of hydroxyl groups on the quassinoid skeleton generally results in an altogether structurally highly diverse class of natural products. As a result of this huge diversity, a plethora of promising bioactivities has been described up to today: anti-cancer, cytotoxic, phytotoxic as well as antiparasitic properties, only to name a few [16,17,18,19].

Only recently, the specific anthelmintic potential of the two quassinoids ailanthone (from *A. altissima*, Simaroubaceae) and bruceine A (from *Brucea javanica,* Simaroubaceae) has been described against the model organism *Caenorhabditis elegans*, resulting in the infertility of *C. elegans* individuals treated with the mentioned quassinoids without affecting the overall life expectancy of the treated worms [6]. This specific infertility effect was observed in L4 hermaphrodites and moreover specified to be irreversible [6]. AFM-based ultrastructural elucidations of ailanthone-treated gonadal tissues carried out by Lehmann et al. on *C. elegans* ultra-sections additionally indicated morphological impairments in the gonadal tissues. Based on these preliminary investigations, the present study aimed at the conformation of the mentioned results on ailanthone against *C. elegans* as well as on the identification of a potential anthelmintic mode of action of both nematicidal quassinoids by means of advanced light microscopic assessments as well as by Atomic Force Microscopy (AFM)-based ultrastructural evaluations of quassinoid-treated compared to untreated *C. elegans* morphology.

The elucidation of ultrastructural data of internal organs of *C. elegans* is altogether a quite challenging task. Commonly used techniques such as Transmission Electron Microscopy (TEM) suffer from certain limitations such as the intense specimen preparation employing highly toxic chemicals, the reduced ability to perform immunostaining procedures on the sample preparations, or the overall needed costs for maintenance of the rather complex electron microscope machinery. Therefore, we used within the following study a newly developed, facile, and economic technique [20], allowing the rapid identification of specific tissues in immobilized *C. elegans* ultra-sections by epifluorescence microscopy and subsequent ultrastructural data acquisition on those tissues by AFM. This new protocol enabled us to identify and to nanoscopically characterize specific tissues of the reproductive system of *C. elegans* such as the gonads or the rather small spermathecae by AFM with high efficiency. This subsequently led to the identification of specifically localized ultrastructural defects induced in the reproductive tissues of *C. elegans* by quassinoid treatment and allowed pinpointing an apoptosis-like mechanism of action concerning the infertility effect of the tested quassinoids against *C. elegans*.

## 2. Results

### 2.1. (Ultra)-Structural Investigations of C. elegans Morphology via Light and Atomic Force Microscopy

In the presented study, the morphology of the germ line tissues of quassinoid-treated (ailanthone and bruceine A) and untreated *C. elegans* L4 hermaphrodites was assessed using three different microscopic techniques: DIC, epifluorescence microscopy, and ultrastructural AFM.

### 2.2. Differential Interference Microscopy (DIC)

**Figure 2:**
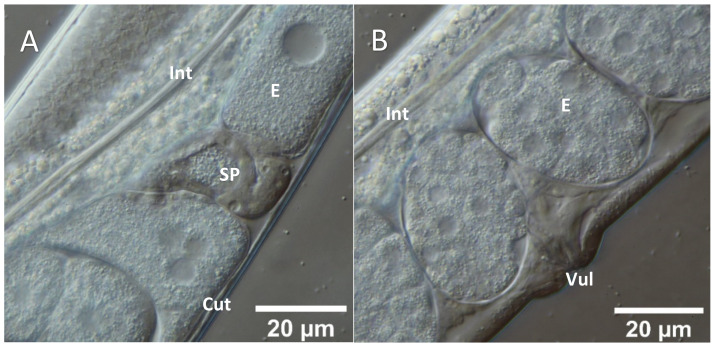

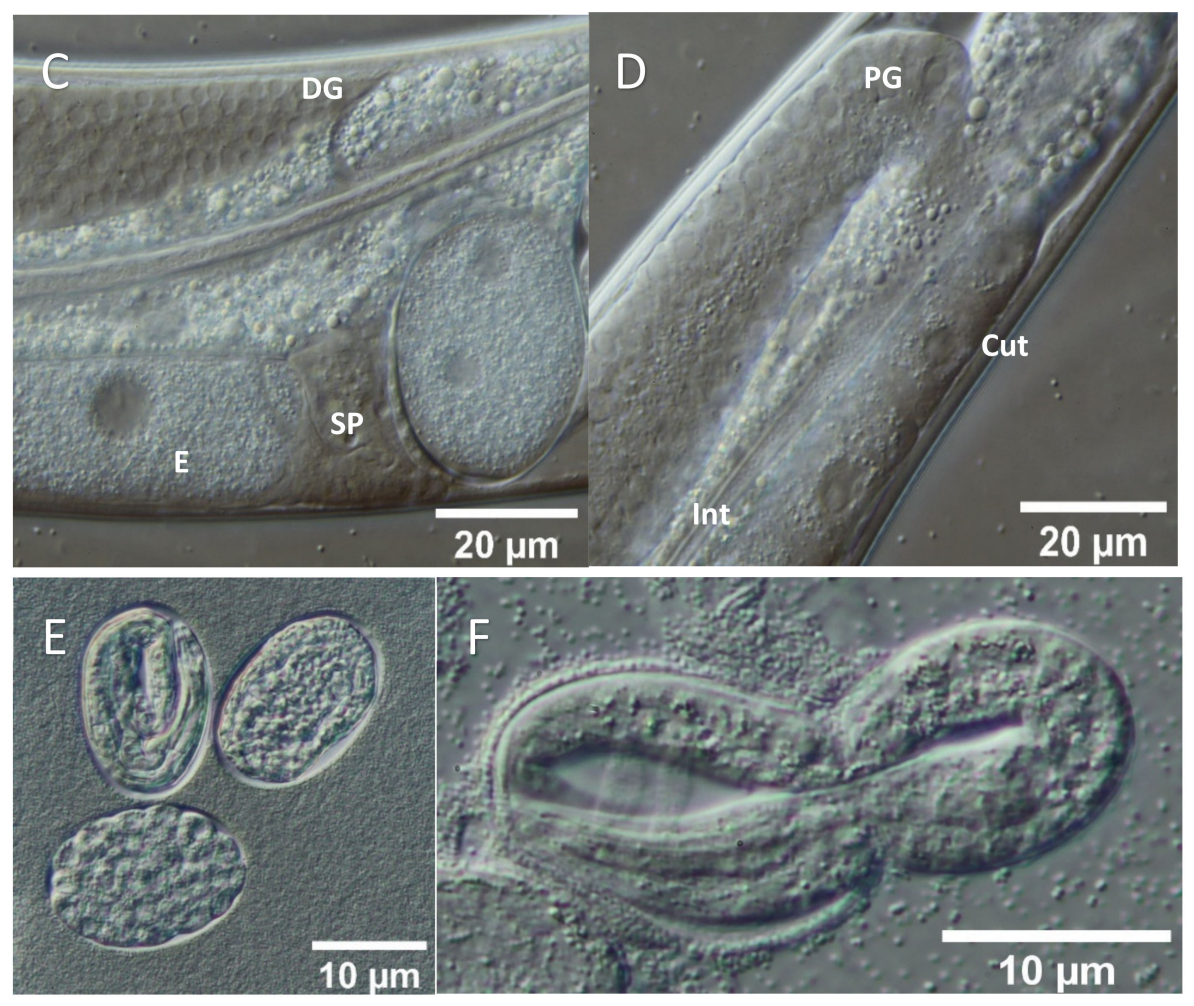
Germ line morphology of untreated L4 WT *C. elegans* hermaphrodites elucidated by Differential Interference Microscopy (DIC). (**A**): Representative spermathecal region surrounded by natively developing embryos (**B**): Representative vulvar region surrounded by developing embryos (**C**): Close up on distal gonadal arm (**D**): Close up on proximal gonadal arm (**E**,**F**): Typical physiological stages of embryo development in an untreated individual. Cut = cuticle, DG = distal gonad, E = egg, Int = intestine, PG = proximal gonad, SP = spermatheca, Vul = vulva.

**Figure 3:**
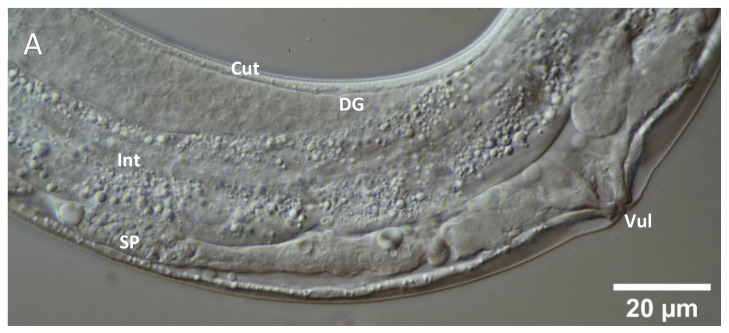

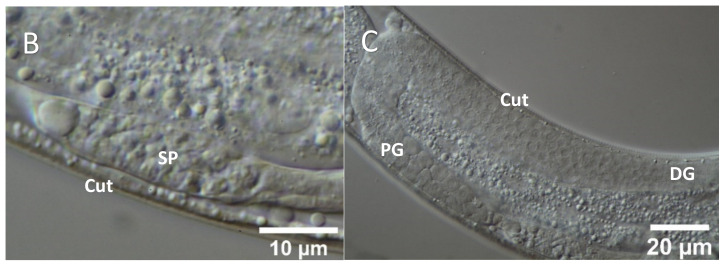
Germ line morphology of bruceine A-treated (100 µM, 48 h) L4 WT *C. elegans* hermaphrodites elucidated by Differential Interference Microscopy (DIC). (**A**): Representative DIC close up on vulvar region of a bruceine A-treated L4 hermaphrodite; note the overall missing embryos in the uterus region (**B**): Representative close up on spermathecal region of a bruceine A-treated *C. elegans* hermaphrodite; specific morphological impairments were not clearly recognizable by DIC microscopy in single spermathecae. (**C**): Gonadal arm of bruceine A-treated *C. elegans* individual; specific morphological impairments were not clearly recognizable by DIC microscopy. Cut = cuticle, DG = distal gonad, Int = intestine, PG = proximal gonad, SP = spermatheca, Vul = vulva.

As an initial step of the assessment of potential structural defects induced by the quassinoid treatment, treated and untreated L4 hermaphrodites were visualized using DIC microscopy as a standard technique for light microscopic investigations of *C. elegans*. Summarizing, L4 individuals treated for 48 h with ailanthone and bruceine A (c _ailanthone_ = 100 µM, c _bruceine A_ = 50 µM) showed distinctive defects in the germ line tissues compared to the respective untreated L4 hermaphrodites (Figure 2 and Figure 3). Especially, an impaired development and an overall reduced amount of embryos in the treated individuals were detectable by DIC microscopy (Figure 3). Additionally, the morphology of single embryos was also altered by the quassinoid treatment, resulting e.g., in untypical overall shapes or decreased visible cellularization as well as missing clear differentiation between single embryos. Moreover, DIC microscopy clearly indicated a fatal developmental delay of oocytes in the proximal gonads (Figure 3). In contrast to that, DIC microscopy did not show any structural abnormalities in the rachis, the body muscle cells, the enterocytes, or in other tissues of *C. elegans* individuals (data not shown). Altogether, the structural defects evoked by ailanthone (data not shown) as well as by bruceine A (Figure 3) treatment and elucidated by DIC microscopy were highly comparable.

### 2.3. Acridine Orange (AO)-Based Evaluation of the Nucleic Acid Composition and Distribution in Single Cells of the C. elegans’ Reproduction System by Epifluorescence Microscopy

**Figure 4:**
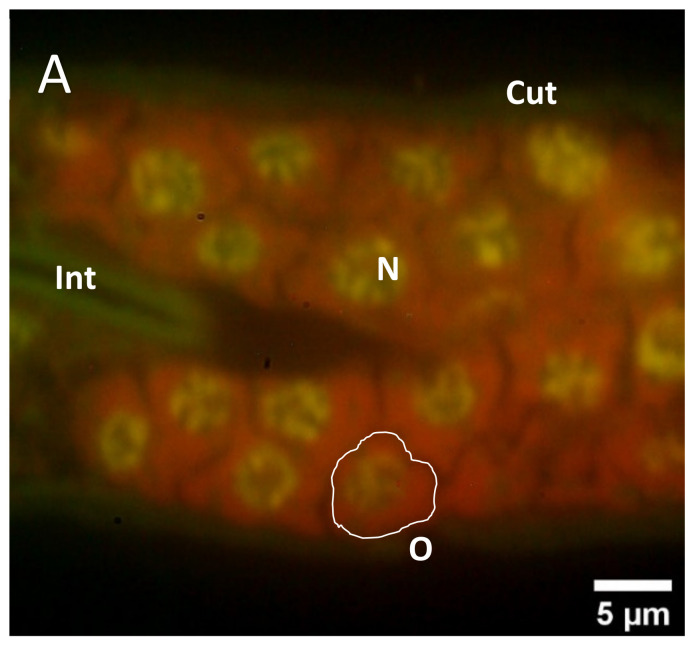

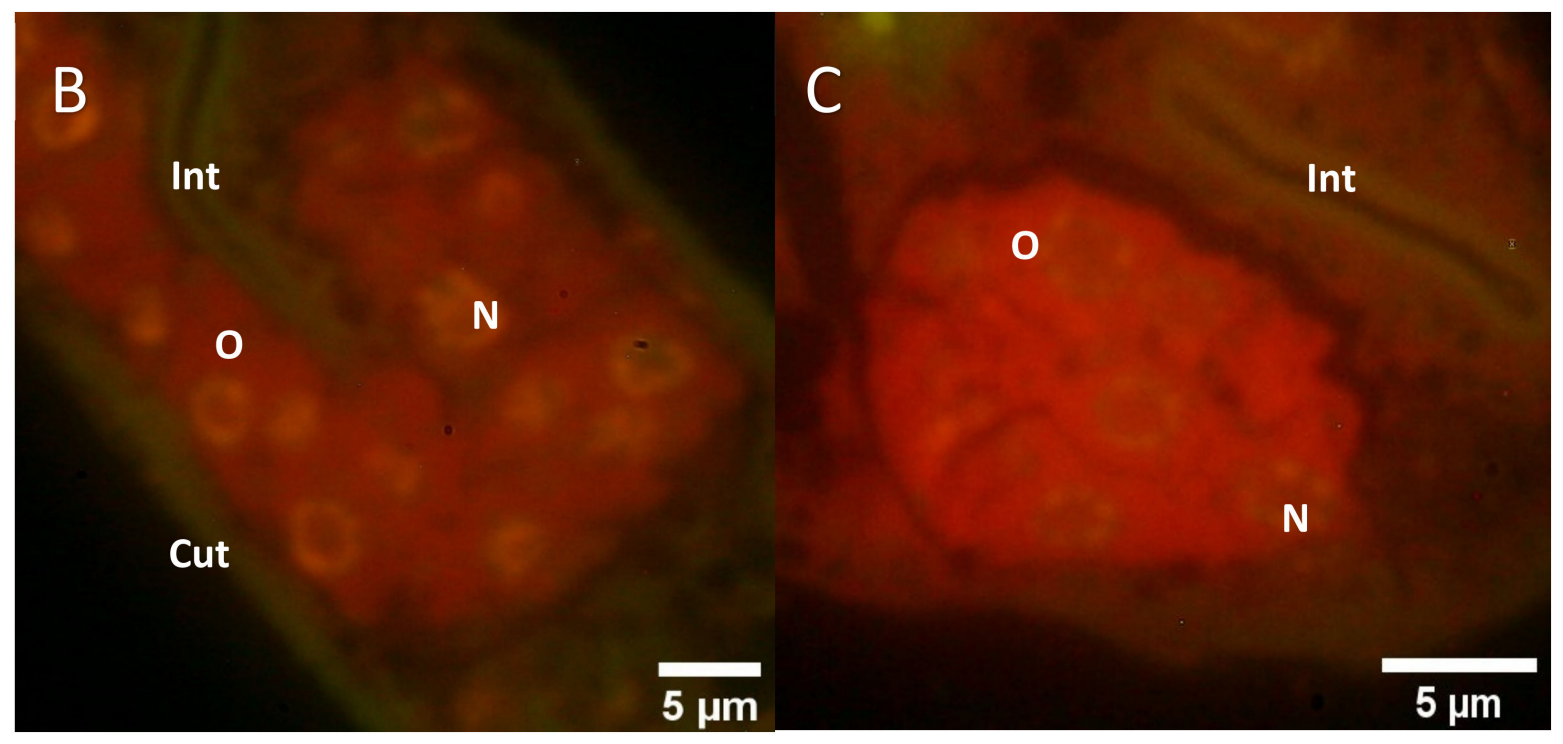
High-magnification epifluorescence images of gonadal tissues in acridine orange-stained ultra-sections of quassinoid-treated as well as untreated L4 *C. elegans* individuals. (**A**): Representative depiction of untreated gonadal tissue after AO staining; note the prominent yellow-green fluorescence in nuclei of single oocytes (**B**): Representative depiction of ailanthone-treated gonadal tissues after AO staining; note the reduced fluorescence signal in the nuclei as well as the overall increased red fluorescence in the attendant cytoplasm (**C**): Representative depiction of brucein A-treated gonadal tissues after AO staining; note the change in overall fluorescence similar to ailanthone-treated gonadal tissues (**B**). Cut = cuticle, Int = intestine, N = nucleus, O = single oocyte.

**Figure 5:**
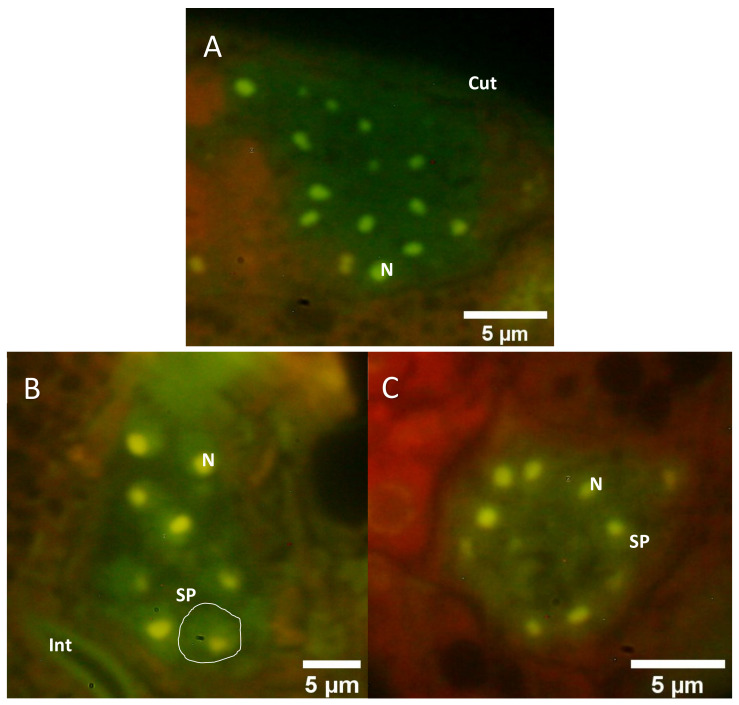
High-magnification epifluorescence images of spermathecal tissues in acridine orange-stained ultra-sections of quassinoid-treated as well as untreated L4 *C. elegans* individuals. (**A**): Representative depiction of untreated spermathecal tissues after AO staining. (**B**): Representative depiction of ailanthone-treated spermathecal tissues after AO staining; note the inhomogeneous green fluorescence in single cytoplasmic areas as well as the overall increased granularity of the tissue (**C**): Representative depiction of brucein A-treated spermathecal tissues after AO staining; note the inhomogeneous green fluorescence in single cytoplasmic areas as well as the overall increased granularity of the tissue. Cut = cuticle, Int = intestine, N = nucleus, SP = single spermatocyte.

In order to allow more specific insights into the detailed structure of germ line tissues of quassinoid-treated versus untreated *C. elegans* hermaphrodites, PEG-embedded specimen were ultra-sectioned, immobilized, stained with the fluorescent dye acridine orange, and subsequently analyzed by epifluorescence microscopy. Due to the fact that the AO-dye is intercalating mainly with nucleic acids [21], information about the respective nucleic acid conditions and the distribution within the cells or tissues was obtained by epifluorescence microscopy, employing excitation at *λ* = 466 nm. In ultra-sections of untreated individuals, the AO-stained reproductive tissues were clearly distinguishable from each other, e.g., gonadal tissues emitting red fluorescence including brightly shining green fluorescent circles along the nucleus´ perimeter in single germ cells (Figure 4A), whereas the spermathecae appeared in a homogenously green fluorescent light and contained bright green spots, which are corresponding to the nuclei of spermatids and spermatocytes (Figure 5A). Additionally, gonadal tissues typically exhibited clear cell borders between individual oocytes. Moreover, a homogenous red fluorescence was emitted from the cytoplasm of single oocytes. In case of the untreated spermathecal tissues, intense green fluorescence was emitted by single nuclei accompanied with typically homogenously distributed green fluorescence within the cytoplasm of individual spermatids and spermatocytes.

Concerning the ailanthone-treated gonadal tissues, obvious structural abnormalities could be detected by AO-mediated epifluorescence microscopy. Typically, clear cell borders between single oocytes were not observable after ailanthone treatment (Figure 4B). Additionally, the fluorescent signal emitted by the nuclear regions of single oocytes was also impaired, showing far lower fluorescence compared to nuclear regions of untreated oocytes. In case of bruceine A-treated gonadal tissues, highly comparable effects to the ailanthone treatment were observed via epifluorescence microscopy (e.g., loss or reduction of clear cell borders between single oocytes as well as impaired nuclear fluorescence compared to untreated control tissues). Interestingly, the fluorescence signal from the cytoplasm of quassinoid-treated oocytes was overall more intense compared to the signal from untreated gonadal tissues.

Concerning the quassinoid-treated spermathecal tissues, specific morphological alterations were also detected by AO-mediated epifluorescence microscopy. Primarily, the distribution of AO fluorescence in the cytoplasm of single spermatids or spermatocytes did not show a homogenous distribution as compared to untreated spermathecal tissues (Figure 5B,C). Additionally, increased vacuolization within the spermathecal tissues was also observed via epifluorescence microscopy, indicating specific cellular defects of the investigated quassinoid-treated spermathecae.

Apart from the mentioned impairments detected in the germ line tissues of *C. elegans*, no morphological alterations were recorded in non-reproductive tissues.

### 2.4. Analysis of the Ultrastructural Morphology after Quassinoid Treatment by AFM

In order to assess the alterations observed by DIC and epifluorescence microscopy in ultrastructural detail, nanoscopic data acquisition was carried out by AFM via tapping mode imaging in air on immobilized ultra-sections of treated and untreated *C. elegans* L4 hermaphrodites.

Based on the epifluorescence-mediated identification of gonadal and spermathecal tissues in single ultra-sections, precise positioning of the measurement probe and subsequent AFM data assessment on a high number of individuals and corresponding germ line tissues became easily possible by coupling an inverted epifluorescence microscope to an AFM. This subsequently allowed the reliable ultrastructural assessment of treated as well as untreated germ line morphology. Without the mentioned correlation of epifluorescence microscopy to AFM, the specific identification of the rather small spermathecae by AFM alone proved to be tedious and time consuming.

**Figure 6:**
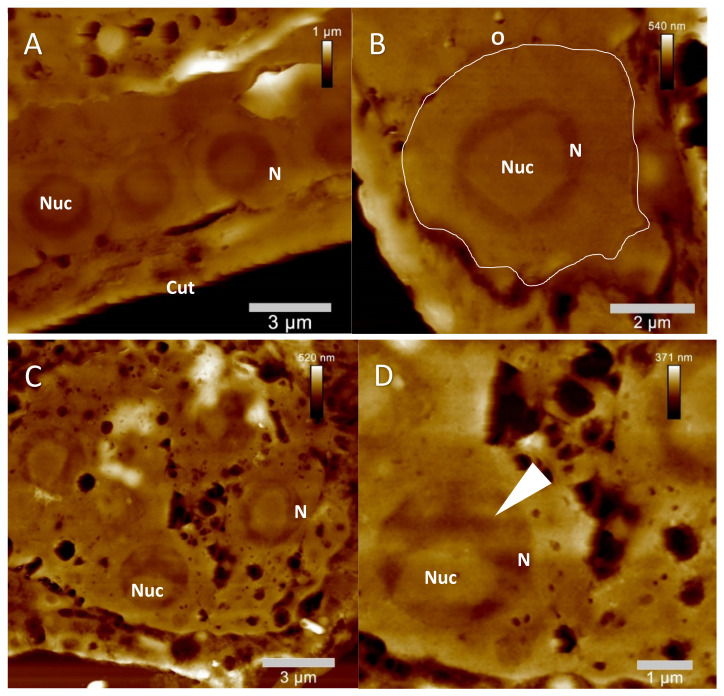

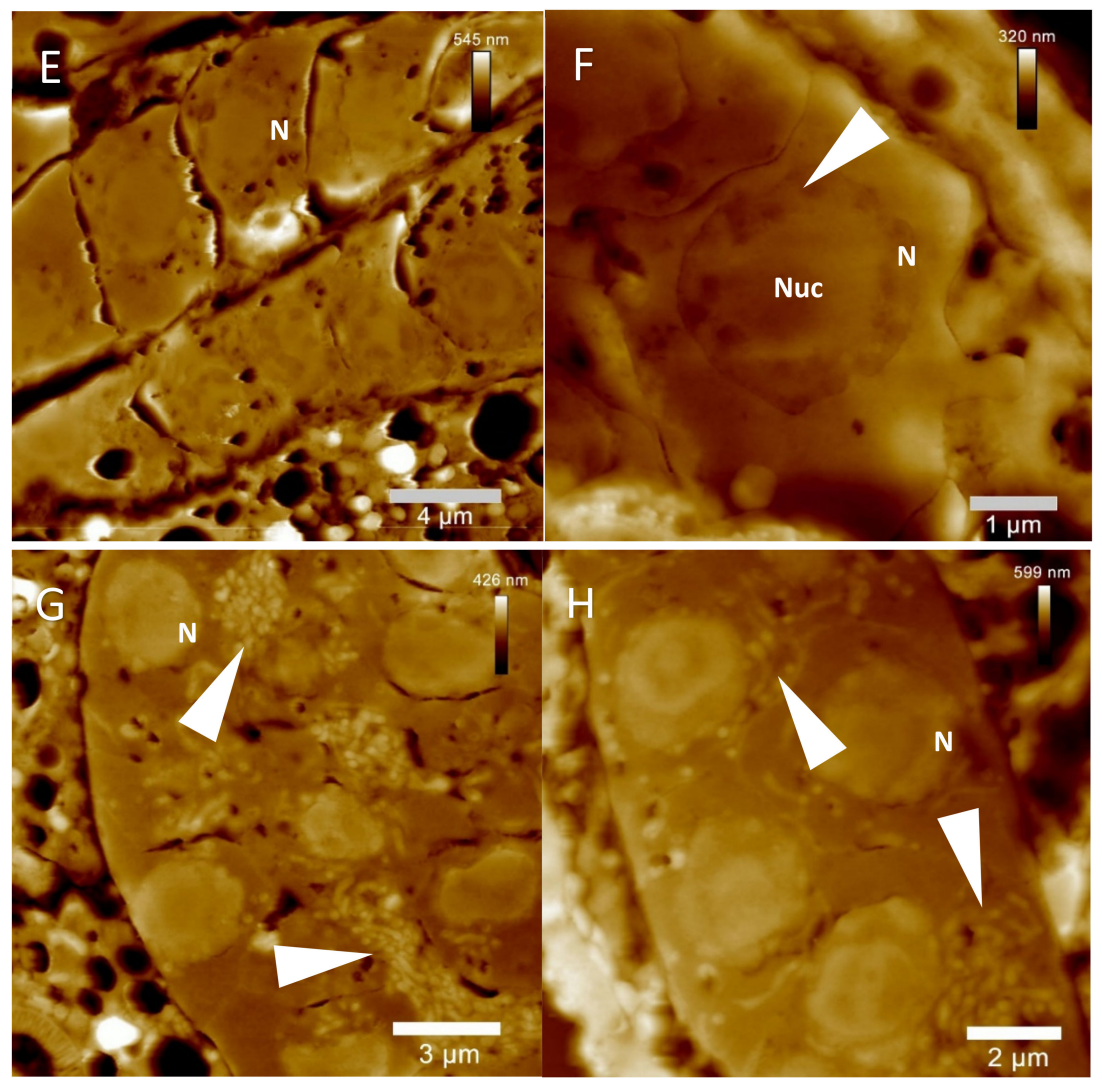
AFM-elucidated ultrastructure of *C. elegans* L4 hermaphrodite´s gonadal tissues after treatment with the quassinoids ailanthone (c _ailanthone_ = 50 µM) and bruceine A (c _bruceine A_ = 100 µM) and compared to untreated gonadal morphology. (**A**): Representative native gonadal tissue, single oocytes, and corresponding nuclei are easily detectable (**B**): Close up on a single untreated oocyte (**C**): Representative gonadal tissue of ailanthone-treated individual (**D**): Close up on single ailanthone-treated oocyte; note the specific vacuolization in the gonadal tissue as well as the impaired morphology of nucleus and nucleolus (e.g., highly condensed chromatin (white arrowhead) compared to untreated karyoplasm morphology (**A**,**B**)) (**E**): Representative gonadal tissue of bruceine A-treated individual (**F**): Close up on single bruceine A-treated oocyte; note the increased vacuolization in the gonadal tissue as well as the impaired morphology of nucleus and nucleolus (e.g., highly condensed chromatin (white arrowhead) compared to untreated karyoplasm morphology (**A**,**B**)) (**G**): Depiction of ailanthone-treated gonadal region in a L4 hermaphrodite, showing the accumulation of mitochondria (white arrowheads) most probably in the rachis region. (**H**): Close up on gonadal region in a L4 hermaphrodite after ailanthone treatment showing the accumulation of mitochondria (white arrowheads) most probably in the rachis region. Cut = cuticle, N = nucleus, Nuc = nucleolus, O = single oocyte.

The ultrastructural morphological composition of reproductive tissues in untreated *C. elegans* individuals altogether showed germ cells and oocytes with clearly delineated cell outlines to one another (Figure 6A,B). Moreover, the integrity of single germ cell membranes as well as the overall shape of the oocytes was clearly preserved as could be expected from untreated *C. elegans* morphology. Furthermore, nuclei appeared to be located in the middle of matured oocytes, containing a single nucleolus and showing overall homogenous nuclear membrane integrity (note that borders from nuclei as well as nucleoli appeared to be of a rather round and smooth shape, Figure 6A,B). Typically, the nucleoli were evenly distributed within the corresponding nuclei. Additionally, the gonadal tissues appeared to be altogether separated from the surrounding organs or cells by a clearly identifiable basal membrane. In summary, the expected nuclear morphology of healthy gonadal tissues of *C. elegans* was in accordance with the available ultrastructural literature data [22,23,24].

In case of the quassinoid-treated gonadal tissues, ultrastructural data acquisition by AFM allowed a more detailed insight into the treatment-induced morphological alterations. Both ailanthone as well as bruceine A treatment provoked distinctive ultrastructural defects in the gonadal tissues in comparison to the untreated control sections, such as increased vacuolization, less obviously detectable oocyte membranes, as well as characteristic alterations of the nucleoplasm and of the corresponding nucleoli regions (Figure 6C–F). Increased vacuolization did develop to a comparable extend in ailanthone as well as in bruceine A-treated gonads. This alteration became especially apparent due to the fact that these vacuole-like cavities were only very scarcely observed in untreated gonadal tissues (Figure 6A,B). In contrast to the untreated morphology, cell borders of the oocytes were typically invisible in quassinoid-treated gonadal tissues. Additionally, characteristic ultrastructural alterations were also induced in the nuclear regions of gonadal tissues by quassinoid treatment. In contrast to the morphological details of untreated single oocytes, quassinoid-treated gonads exhibited apparent changes in ultrastructural morphology such as the specific introduction of agglomerations to the nucleoplasm of oocytes, the loss of a smooth morphology of their nuclear membrane, as well as specific changes in the overall morphology of the oocytes´ nucleoli (e.g., loss of smooth edges, Figure 6C–F).

**Figure 7:**
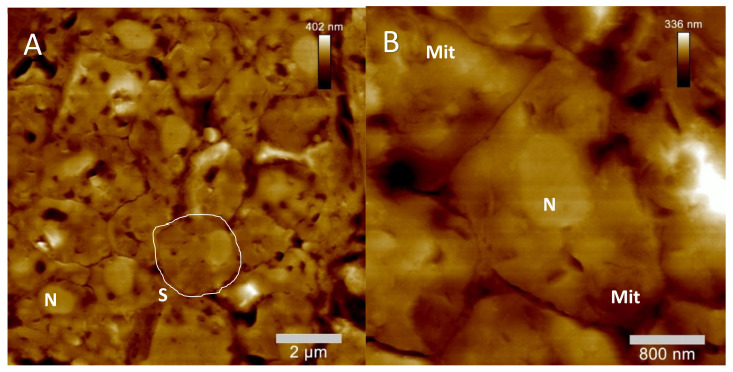

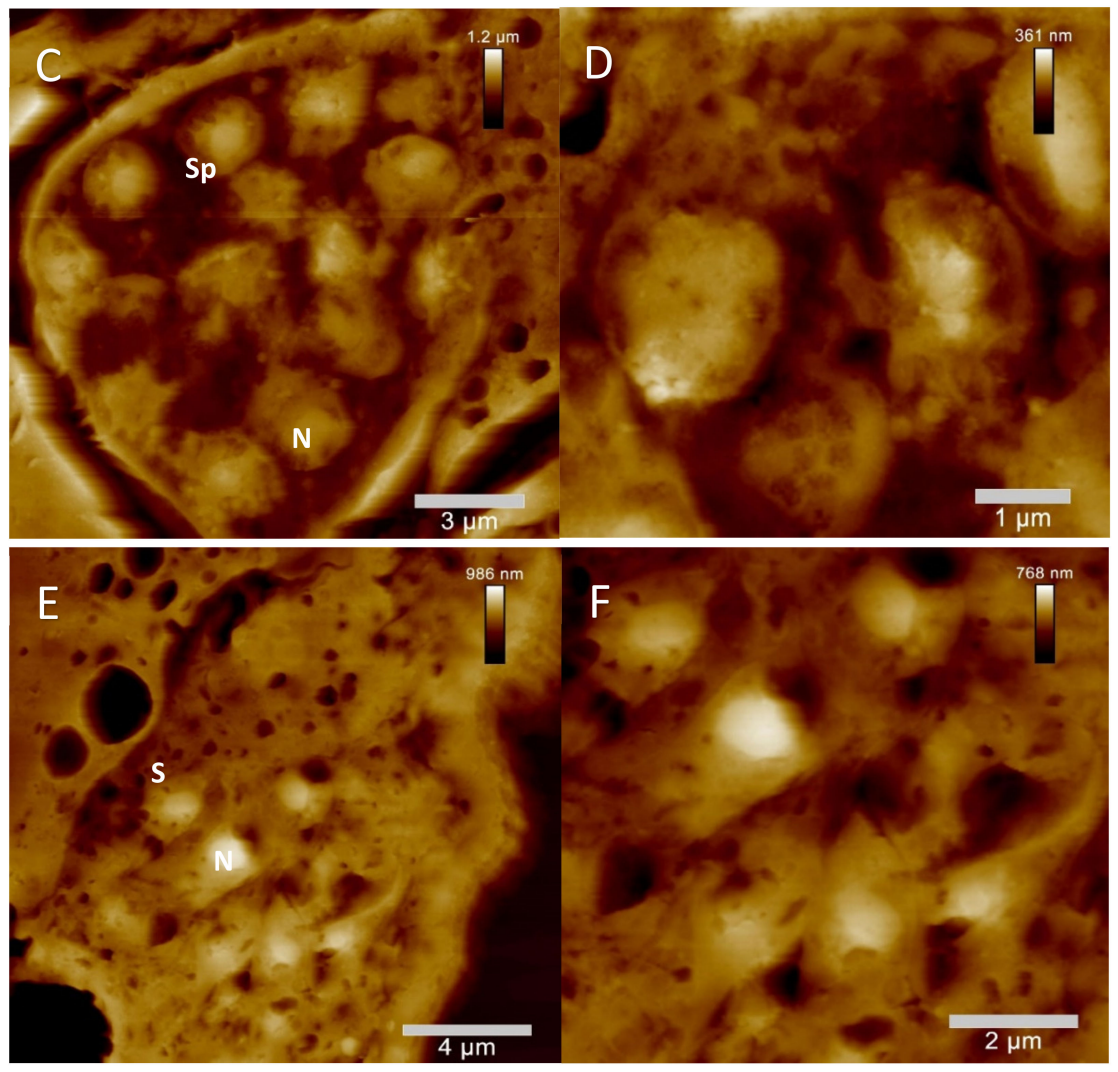
AFM-elucidated ultrastructure of *C. elegans* L4 hermaphrodite´s spermathecal tissues after treatment with the quassinoids ailanthone (c_ailanthone_ = 50 µM) and bruceine A (c_bruceine A_ = 100 µM) and compared to untreated spermathecal morphology. (**A**): Representative untreated spermathecal tissue (**B**): Close up on single spermatocytes (**C**): Representative ailanthone-treated spermathecal tissue (**D**): Close up on single spermatid-like cells (**E**): Representative bruceine A-treated spermathecal tissue (**F**): Close up on single spermatocytes. Mit = mitochondrium, N = nucleus, Nuc = nucleolus, S = single spermatocyte, Sp = single spermatid.

In case of the AFM-based data acquisition on sectioned spermathecal tissues, untreated control sections showed ultrastructural morphology in accordance with the available literature on spermathecal tissues of *C. elegans* [22]. Single spermatids, including their cell membranes as well as their corresponding nuclei, were easily detectable by intermittent contact AFM in air, showing clearly delineated cellular borders as well as easily detectable nuclear regions (Figure 7A,B). Additionally, the location and morphology of mitochondria within single spermatocytes was detectable by AFM analysis of ultra-sectioned spermathecal tissues (Figure 7B).

In case of the quassinoid-treated spermathecal tissues, the typical morphology of single spermatids within spermathecal tissues was altogether largely altered. Concerning the ailanthone-treated samples, single spermatids were detectable within spermathecal tissues but showed apparent changes in their ultrastructural composition. Mainly, the nuclear membrane clearly detectable in untreated spermatids (Figure 7A,B) was typically unrecognizable in ailanthone-treated spermathecal tissues. Additionally, single mitochondria could not be detected in ailanthone-treated spermatids. Although single spermatids were clearly identified in spermathecal tissues treated with ailanthone, their overall morphology was obviously impaired, sometimes leading to the evident loss of cellular borders within single spermatids (Figure 7D) as well as the loss of overall spermathecal tissue integrity.

For the bruceine A-treated spermathecal tissues, obvious impairments of the ultrastructural morphology and composition were detected by AFM again. As was the case for ailanthone-treated spermathecal tissues, bruceine A treatment led to the obvious loss of membrane integrity of single spermatids. Additionally, the overall morphology of the nuclear membranes was also typically disrupted. Moreover, the easily recognizable mitochondria of untreated spermatocytes were again not identified in bruceine A-treated spermathecal tissues. In contrast to ailanthone-treated spermathecae, an increased vacuolization was additionally recognized compared to untreated controls.

In summary, ultrastructural AFM data acquisition on single ultra-sections from untreated as well as from quassionid-treated *C. elegans* individuals led to the identification of characteristic morphological impairments specifically located in germline tissues. The mentioned alterations were overall not identified in non-germline tissues (e.g., enterocytes or body muscle cells) by any of the mentioned microscopic techniques.

## 3. Discussion

The presented study aimed at the detailed investigation of morphological defects of reproductive tissues in L4 *C. elegans* hermaphrodites after treatment with two quassinoids (ailanthone and bruceine A) in order to potentially pinpoint their anthelmintic mode of action.

Initial DIC investigations carried out during this study already proved significant structural alterations induced in germ line tissues of L4 *C. elegans* hermaphrodites after quassinoid treatment compared to untreated individuals. In case of both quassinoids, observed effects included the decreased development of proximal gonads (e.g., undifferentiated embryos) as well as a significantly reduced amount of meiotic germ cells compared to the untreated control experiments (Figure 3).

By employing the easily allocatable fluorescent AO dye on ultra-sections of *C. elegans* for the first time, valuable insights into the nucleic acid distribution of cells of the reproductive system of quassinoid-treated *C. elegans* individuals were obtained. More detailed, epifluorescence microscopy of untreated AO-stained ultra-sections from L4 hermaphrodites showed brightly shining green fluorescent signals within nuclei of single germ cells most probably indicating the interaction between AO and double-stranded DNA (Figure 4A). In contrast to that, AO-stained ultra-sections of ailanthone and bruceine A-treated L4 larvae showed significantly less brightly shining green fluorescence accompanied by additional orange fluorescence signals in the nuclei of germ cells compared to untreated control germ line tissues (Figure 4B,C). An assumable interpretation of the mentioned change from a green to a significantly more orange fluorescence signal in nuclei from treated oocytes could be given by an alteration in the nucleic acid composition by means of loss or defects within double-stranded DNA caused by quassinoid treatment, probably indicating apoptosis-like defects. Furthermore, untreated as well as treated gonads were altogether emitting red fluorescence within their cytoplasm. Informatively, the germline is the only proliferative tissue in L4 *C. elegans* hermaphrodites and therefore has a considerable amount of mRNA attendant in the cytoplasm of germ cells and the rachis [25], resulting in all-over homogenously red fluorescent gonadal tissues after AO staining. Interestingly, these findings were also indicating some changes within the nucleic acid composition of investigated gonadal nuclei in treated individuals (Figure 4B,C), which might be explained by the overall reduction of present DNA in the nuclei induced by quassinoid treatment. It could already be concluded from these light microscopic investigations that the quassinoids ailanthone and bruceine A most probably have a genotoxic potential against *C. elegans* reproductive tissues in general, which is most likely due to their interaction potential with nucleic acids.

In order to more thoroughly assess a potential anthelmintic mechanism of action of the investigated quassinoids, AFM as an ultrastructural technique allowing the nanoscopic depiction of the mentioned defects in the *C. elegans* germ line was subsequently applied to AO-stained ultra-sections. Mediated by the facile identification of gonadal and spermathecal tissues by AO epifluorescence microscopy, a high number of treated and untreated germ line tissues could be easily assessed by ultrastructural AFM for the first time, allowing the more detailed interpretation of morphological alterations induced by quassinoid treatment.

The application of the mentioned innovative AFM technique led to the specific identification of ultrastructural alterations induced by quassinoid treatment in the gonadal tissues of *C. elegans* e.g., underdeveloped oocytes surrounded by highly vacuolized gonadal tissues, a generalized decrease in germ cell cytoplasm, and the characteristic condensation of nuclei regions within single oocytes (Figure 6C–F). Interestingly, the mentioned alterations evoked by quassinoid treatment were overall highly comparable to the ultrastructural characteristics appearing during physiological apoptosis in germ line cells of *C. elegans* reported in the available literature [26], most probably indicating an apoptose-like effect induced by quassinoid treatment to the gonads of *C. elegans*.

Furthermore, another interesting ultrastructural effect elucidated in treated gonadal tissues within the study at hand also indicated potential apoptotic changes to oocytes induced by quassinoid treatment: Raiders et al. identified mitochondrial export from germ cells into the commonly shared cytoplasm of the syncytium (rachis) before the development of further apoptotic characteristics [22,27]. As depicted in Figure 6G,H, ailanthone-induced “agglomerations” within the rachis and germ cells showed altogether similar morphologies compared to TEM data from Raiders et al. [27]. Judging from the dimensions of single particles of the mentioned agglomerations (Figure 6G,H) as well as from the data reported by Raiders et al., it seems feasible to consider them to be mitochondria involved in a comparable mechanism as observed by Raiders et al. Without being able to directly identify the mentioned agglomerations from the AFM data alone, it still can be postulated that some specific “recycling mode” of the germ cells comparable to the findings by Raiders et al. is induced in gonads of L4 *C. elegans* hermaphrodites by ailanthone treatment.

Concerning the ultrastructurally investigated defects in the spermathecae of quassinoid treated ultra-sections of *C. elegans* within the presented study, striking morphological defects of those tissues were observed after treatment with ailanthone and bruceine A compared to untreated individuals (Figure 7A–F, [22,28,29]). In the case of ailanthone-treated *C. elegans* individuals, obvious morphological alterations compared to untreated as well as bruceine A-treated worms were identified by ultrastructural AFM (e.g., overall round-shaped male germ cells with clearly visible cell borders, cell debris, and tissue interruptions within the spermathecae as well as abundant agglomerations). It is interesting to note that ailanthone-treated germ cells showed similarly formed round shapes when compared to the available TEM data of spermatid morphology from the literature [22,28,29]. Therefore, it is suggestable that ailanthone might inhibit cell differentiation during the spermatogenesis especially during the development from spermatids to mature spermatocytes. In contrast to ailanthone-treated spermathecae, bruceine A-treated individuals showed differing morphological alterations (e.g., single germ cells within the spermathecae merging into one another and showing no separate cell borders as well as the occurrence of condensed areas within the spermathecae). These findings could indicate cell defects of mature spermatocytes that specifically appeared after treatment with bruceine A. Additionally, in some of the bruceine A-treated individuals of the study at hand, no sperm could altogether be observed in some spermathecae. This defect could not be clearly correlated with the available ultrastructural data from the literature, but it is assumable that bruceine A treatment might have triggered critical defects in male germ cells, leading to empty spermathecae. Altogether, quassinoid treatment may have triggered genotoxically induced DNA damages in proliferative meiotic male germ cells, subsequently resulting in apoptosis-like effects and sometimes empty spermathecae.

Regarding the morphological defects determined in the study at hand, the identification of an anthelmintic mode of action of ailanthone and bruceine A concerning the reproduction system of *C. elegans* was narrowed down to a potential non-physiological apoptosis-like mechanism by the use of a variety of microscopic techniques. In terms of the highly related chemical properties of ailanthone and bruceine A, it is likely to postulate that both quassinoids exert similar modes of action against *C. elegans*. A plausible theory for the different morphological findings within bruceine A and ailanthone-treated individuals (e.g., spermathecal tissues) could be attributed to differing solubilities as well as to the probably different biological effectiveness of the two investigated quassinoids.

Due to the ultrastructural preservation of the native morphology of non-reproductive tissues and organs of treated individuals (e.g., cuticle, pharynx, intestine or body muscle cells, showing altogether unimpaired nuclear and nucleoli morphology such as the gonadal cells in Figure 6A,B) as well as due to the resulting infertility of treated worms, it seems feasible to cautiously assume that ailanthone and bruceine A are mainly targeting reproductive tissues in *C. elegans*. Due to this mechanism of action, targeting *C. elegans* gonadal tissues in general and subsequently leading to infertile worms, quassinoids, and their exerted mode of action might be of relevance for the development of innovative anthelmintic therapies.

On the other side, a pharmaceutical development of quassinoids as deworming drugs in livestock or in human medicine has also to be aware of the potential toxicity of these diterpenes on the host. Especially DNA damage and the subsequent induction of apoptosis by ailanthone has to be taken into account [11,30], and systematic toxicological studies have to be performed to clarify the risk–benefit ratio. Preclinical toxicology and toxicokinetic data in mice indicated dose-dependent (2.5 to 10 mg/kg) organ toxicity, with stomach and intestinal tissues being the main target organs [31].

To continue the development of quassinoid-based anthelmintics, future investigations should analyze a broader spectrum of available quassinoids concerning their structure–activity toxicological relationships against *C. elegans* as commonly employed model organism for the development of anthelmintic drugs [32] and additionally against pathogenic worm species. Further steps to narrow down the potential anthelmintic mode of action of ailanthone and bruceine A could also be conducted by specific genome analyses (e.g., egl-1 [26,33,34], cep-1 [35]), by analysis of gene expression levels or by proteome-based investigations (e.g., ced-3 protein as a major component of programmed cell death in *C. elegans* [26,31]).

## 4. Materials and Methods

### 4.1. Inactivation of E. coli (OP50 Strain)

*C. elegans* individuals of this study were fed with heat-inactivated OP50 *E. coli* cells in order to minimize distractions probably caused by living bacterial cells in the bioassay. Initially, suspension cultures of OP50 *E. coli* were incubated overnight (100–200 mL of liquid OP50 medium containing 5 × 10^9^ cells/mL (OD600) agitated at 120 rpm at 37 °C). One mL of the homogenized suspension was afterwards transferred into 2 mL Eppendorf reagent tubes (Eppendorf AG, Hamburg, Germany), and freezing by submersion into liquid nitrogen was performed prior to heating the reagent tubes to 70 °C for 10 min. This process was repeated twice, leading to the inactivation of the bacteria. Centrifugation at 6500× *g* was subsequently performed, the supernatant was removed and discarded, and the resulting pellet was resuspended in M9 medium. As a final step, cholesterol was added to a final concentration of 0.1% before storing the prepared tubes at 4 °C until further use.

### 4.2. Preparation of Nematode Growth Medium (NGM) Agar Plates Containing OP50 E. Coli Bacteria

Prepared NGM agar [36] was heated in a microwave for 20 min until becoming transparent and liquid. After cooling of the NGM agar down to roughly 55 °C, supplements were added under sterile conditions. The resulting viscous liquid was filled in Petri dishes and allowed to dry for at least 3 h under ambient conditions. Finally, 800 µL of OP50 suspension were added under sterile conditions, and the resulting NGM plates supplied with OP50 bacteria were stored at 4 °C until further use.

### 4.3. Cultivation of C. elegans Individuals

*C. elegans* wild-type (WT) individuals were grown monoxenically at 20 °C on Nematode Growth Medium (NGM) agar plates supplied with inactivated *E. coli* cells (OP50 strain) according to the methodology by Brenner et al. [36]. Every 3 to 4 days, roughly 30–50 individuals were transferred to fresh NGM-OP50 agar plates [36] in order to reduce stress and starvation to a minimum. The agar plates were subsequently stored at 7 °C until further use.

### 4.4. Synchronization of C. elegans Individuals

Synchronous larval stages were yielded by the application of an alkaline bleaching protocol according to Lewis et al. [37]. To do so, individuals were firstly washed of a densely populated WT *C. elegans* agar plate into a 2 mL Eppendorf reagent tube (Eppendorf AG, Hamburg, Germany) and were subsequently centrifuged at 6500× *g* for 1 min at 20 °C. The resulting supernatant was afterwards replaced by fresh M9 medium. This washing procedure was repeated altogether three times. After these three washing steps, the worms were resuspended in 1.5 mL synchronization solution, gently agitated for 7 min, and afterwards centrifuged at 6500× *g* for 1 min at 20 °C. The resulting pellet was washed altogether four times with 1.5 mL of M9 medium. These synchronized worm suspensions were incubated overnight to induce hatching. The hatched L1 larval stages were subsequently transferred to new NGM-OP50 plates and incubated at 20 °C. In order to avoid dehydration of the prepared worm plates, storage took place after sealing the agar plates with parafilm.

### 4.5. Test Substances

Ailanthone was supplied by Sigma-Aldrich (Sigma Aldrich Corp., St. Louis, MO, USA) and used at a concentration of 50 µM for inhibition experiments (according to the reported IC_100_ on reproduction inhibition [6]). Bruceine A was kindly provided by Thomas J. Schmidt (Institute for Pharmaceutical Biology and Phytochemistry, University of Muenster, Muenster, Germany) and used for inhibitory experiments at a concentration of 100 µM corresponding to the experimentally determined IC_100_ values reported for bruceines against helminths in the available literature [17].

### 4.6. C. elegans L4-Larval Assay

Synchronized L4-larvae were incubated together with the corresponding agents (ailanthone, bruceine A) in six-well plates, each containing a total of 5 mL assay volume for a period of 48 h.

### 4.7. Fixation

Fixation of *C. elegans* individuals was carried out in freshly prepared glutaraldehyde solution (4% in H_2_O) at 4 °C overnight.

### 4.8. Dehydration and PEG-Embedding of Whole C. elegans Individuals

In order to obtain AFM-suitable ultra-sections of treated as well as untreated *C. elegans* individuals, samples were embedded in PEG 4000 (Waldeck GmbH & Co KG, Muenster, Germany) employing absolute ethanol as intermedium. Prior to the ethanol dehydration, the fixative was removed by repeated washing of the worm samples in Aqua millipore and subsequent centrifugation at 600× *g* for 10 min (altogether three times, supernatants were discarded). Afterwards, dehydration was performed by the application of a H_2_O:EtOH dilution series (H_2_O:EtOH; 85:15, 25:75, 50:50, 70:30, 95:5, and finally 0:100 (absolute and H_2_O-free EtOH)). Each dilution was applied for at least 10 min under gentle agitation at room temperature, and change of dilutions was performed via centrifugation at 600× *g* for 10 min and subsequent removal of the supernatant. In order to ensure the complete absence of remnant H_2_O in the sample, the absolute EtOH step (0:100) was applied altogether three times. Finally, infusion of PEG 4000 (Waldeck GmbH & Co KG, Muenster, Germany) was carried out by the application of an EtOH:PEG dilution series (EtOH:PEG, 50:50, 0:100 (absolute PEG 4000)) at 64 °C under gentle agitation (at least 10 min for each dilution). The final absolute PEG step was applied at least two times, and change of absolute PEG was performed by centrifugation at 600× *g* employing a rotor pre-heated to 64 °C, subsequent removal of the liquid supernatant, and the application of fresh absolute and molten PEG 4000.

### 4.9. Sample Preparation for Ultra-Sectioning

The supernatant of the last absolute PEG was discarded, and the remaining worm pellet in absolute PEG was subsequently gently homogenized using a single bristle hair. The resulting worms suspended in molten and absolute PEG were afterwards transferred into a BEEM Type 00 capsule. Freshly molten absolute PEG 4000 was added, and the suspended worms were allowed to accumulate in the capsule´s tip by gravity via storing the sample at 64 °C overnight. Afterwards, the embedding medium was solidified at room temperature, and the resulting blocks were removed from the capsules. In order to allow mounting of the blocks in the employed ultra-microtome (Reichert Ultracut E, Reichert/Leica, Leica Microsystems, Wetzlar, Germany), a small aluminum rod was melted onto the block´s base.

### 4.10. Ultra-Sectioning and Immobilization of the Sections

Ultra-sectioning of the PEG-embedded specimen was carried out on a Reichert/Leica Ultracut E microtome (Leica Microsystems, Wetzlar, Germany) equipped with freshly prepared standard glass knifes (prepared on a LKB Bromma Knifemaker 7801B (LKB-Bromma, Stockholm, Sweden)). Sections of 350 nm thickness were repeatedly cut from the PEG blocks (sectioning speed around 50 mm/s, inclination angle of 2–3°), yielding easily transferrable sectioning bands. Around 200 sections were transferred as a single band into a droplet of 40 µL aqua Millipore on a poly-l-lysine coated glass slide (Menzel, Braunschweig, Germany). A coverslip was afterwards gently applied, and the resulting covered glass slide was allowed to rest for at least 10 min in order for the ultra-sections to adhere to the coated glass slide. Afterwards, the coverslip was removed by placing the sample in a Petri dish and adding Aqua Millipore until the floating cover slip was easily removable using a pair of tweezers.

### 4.11. Acridine Orange Staining

In order to stain the immobilized ultra-sections for fluorescent light microscopy, the glass slides containing the immobilized sections were covered with acridine orange solution (0.1% in Aqua Millipore) for 5 min. Afterwards, the staining solution was removed, and the resulting preparation was washed at least three times with Aqua Millipore to remove the unbound dye completely.

### 4.12. Dehydration

Dehydration of the immobilized ultra-sections was carried out by the application of a continuous air flow (hand bellows) to the glass slide, resulting in the rapid removal of remnant H_2_O in the ultra-sections. Afterwards, the samples prepared in the described way were directly subjected to analysis by fluorescence microscopy or to an AFM-based image acquisition in air.

### 4.13. Light Microscopic Analysis of Whole C. elegans Individuals and Immobilized Ultra-Sections

Differential Interference Contrast (DIC) microscopy was carried out employing a Leitz/Leica Orthoplan microscope equipped with DIC optics (Leica Microsystems, Wetzlar, Germany). Epifluorescence microscopy of immobilized ultra-sections was also carried out with a Leitz/Leica Orthoplan microscope equipped with a 200W HBO-light source and an epifluorescent illuminator using dichroitic filter cubes (A, N2.1, I3, Leica Microsystems, Wetzlar, Germany) as well as a Leitz/Leica planapochromatic oil immersion objective (63x/1.40 PlApo).

### 4.14. Epifluorescence-Based Identification of Specific Tissues for Subsequent AFM Data Acquisition

In order to easily identify specific tissues in single ultra-sections of *C. elegans* individuals prior to AFM data acquisition, a setup of a Zeiss Axiovert 135 inverted fluorescence microscope (Zeiss, Jena, Germany) coupled with a Veeco/Bruker Bioscope I AFM was employed (Bruker, Karlsruhe, Germany).

### 4.15. Atomic Force Microscopy on Immobilized Ultra-Sections

AFM assessments of the immobilized sections were performed in intermittent contact mode (Tapping mode) using a Veeco/Bruker Bioscope (Bruker, Karlsruhe, Germany) equipped with a Nanoscope IIIa controller (Bruker, Karlsruhe, Germany) and n-type silicon cantilevers (HQ:NSC 14/Al BS, nominal tip radius < 10 nm, nominal spring constant of 5 N/m and a typical resonance frequency around 160 kHz, manufactured by µmash, Sofia, Bulgaria). At about 5% below resonance, a free oscillation of roughly 1.5 volts was employed together with amplitude setpoints of around 1 volt, resulting in stable and reproducible imaging while scanning at speeds around 0.5 Hz. Altogether, three replicates were prepared for each sample (untreated control, ailanthone- and bruceine A-treated), and at least three different individuals have been studied in detail for each replicate in order to assure representative data acquisition.

### 4.16. Processing of AFM Data

Processing of AFM data was carried out using the commercial software NanoScope Analysis 1.5 (Bruker, Karlsruhe, Germany). In case of specific scan noise as well as tilt in the image data, flattening operations (typically 0th and 1st order) were performed after image acquisition.

## Data Availability

Imaging data collected in the course of the study at hand are available from the corresponding author on request.

## Sample Availability

Samples of the compound **1** are commercially available. Samples of compound **2** are available from the authors on request.
